# The Impacts of Climatic Factors and Vegetation on Hemorrhagic Fever with Renal Syndrome Transmission in China: A Study of 109 Counties

**DOI:** 10.3390/ijerph16183434

**Published:** 2019-09-16

**Authors:** Junyu He, Yong Wang, Di Mu, Zhiwei Xu, Quan Qian, Gongbo Chen, Liang Wen, Wenwu Yin, Shanshan Li, Wenyi Zhang, Yuming Guo

**Affiliations:** 1 Ocean College, Zhejiang University, Zhoushan 316021, China; jxgzhejunyu@163.com; 2 Chinese PLA Center for Disease Control and Prevention, Beijing 100071, China; 13693013596@163.com (Y.W.); qianquan@aliyun.com (Q.Q.); wenlty@sohu.com (L.W.); 3 Division of Infectious Diseases, Key Laboratory of Surveillance and Early-Warning on Infectious Disease, Chinese Center for Disease Control and Prevention, Beijing 102206, China; mudi@chinacdc.cn (D.M.); yinww@chinacdc.cn (W.Y.); 4 School of Public Health and Social Work, Institute of Health and Biomedical Innovation, Queensland University of Technology, Queensland 4059, Australia; z13.xu@qut.edu.au; 5 Department of Epidemiology and Preventive Medicine, School of Public Health and Preventive Medicine, Monash University, Melbourne 3004, Australia; gongbo.chen@monash.edu (G.C.); shanshan.li@monash.edu (S.L.)

**Keywords:** orthohantavirus, hantavirus disease, risk map, distributed lag non-linear model, meta-analysis

## Abstract

Hemorrhagic fever with renal syndrome (HFRS) is a rodent-borne infectious disease caused by hantaviruses. About 90% of global cases were reported in China. We collected monthly data on counts of HFRS cases, climatic factors (mean temperature, rainfall, and relative humidity), and vegetation (normalized difference vegetation index (NDVI)) in 109 Chinese counties from January 2002 to December 2013. First, we used a quasi-Poisson regression with a distributed lag non-linear model to assess the impacts of these four factors on HFRS in 109 counties, separately. Then we conducted a multivariate meta-analysis to pool the results at the national level. The results of our study showed that there were non-linear associations between the four factors and HFRS. Specifically, the highest risks of HFRS occurred at the 45th, 30th, 20th, and 80th percentiles (with mean and standard deviations of 10.58 ± 4.52 °C, 18.81 ± 17.82 mm, 58.61 ± 6.33%, 198.20 ± 22.23 at the 109 counties, respectively) of mean temperature, rainfall, relative humidity, and NDVI, respectively. HFRS case estimates were most sensitive to mean temperature amongst the four factors, and the lag patterns of the impacts of these factors on HFRS were heterogeneous. Our findings provide rigorous scientific support to current HFRS monitoring and the development of early warning systems.

## 1. Introduction

Hemorrhagic fever with renal syndrome (HFRS) is a rodent-borne infectious disease caused by hantaviruses (family Bunyaviridae). About 90% of the total reported HFRS cases in the world occurred in China [1,2]. The major causative agents of HFRS in China are *Hantaan* orthohantavirus and *Seoul* orthohantavirus, which are associated with *Apodemus agrarius* and *Rattus norvegicus* as their hosts, respectively. It is reported that farmers, soldiers, and rural residents are at higher risk for hantaviruses because of the large population of rodent hosts in their surroundings and their sub-standard housing conditions [3,4]. Humans can be infected through virus contaminated aerosols, foods, or other materials and rodents bites [5,6]. HFRS has a fatality rate of 5~15% and is often accompanied by clinical manifestations, including sudden fever, myalgia with renal impairment, hemorrhages, and cardiac complications [7,8,9]. The risk factors for HFRS are multifaceted and previous studies have reported that climatic factors may play an important role in the transmission of HFRS due to their effects on the rodent hosts [5,10,11], but large-scale studies are very scarce so far. Characterizing the impacts of climatic factors on HFRS at the national level of China is essential for the development of HFRS early warning systems and is of great value for more evidence-based HFRS control decision-making.

Climate variability can influence the human HFRS transmission through bottom-up effects (e.g., affecting food resources and living environment of rodents, which are crucial for their survival or reproduction). Sufficient precipitation can lead to a rapid growth of primary food production and rodent populations bloom with enough food [12,13,14]. It has been reported that annual precipitation and annual mean absolute humidity were positively correlated with HFRS incidence in Changsha City, China [15]. In addition to precipitation and humidity, temperature and multivariate El Niño Southern Oscillation index (MEI, regarded as a global climatic index) were found to be associated with HFRS transmission in Elunchun and Molidawahaner, China [5]; meanwhile, maximum temperature and southern oscillation index (SOI) were related to the HFRS in Heilongjiang Province, China [16].

Except for climate variability, land cover, and land use types can also impact the rodent populations. Generally, rodents prefer to live in shrubs, grassland, or chaparral with abundant green plants, where sufficient food is provided for their growth [17]. Vegetation index (normalized difference vegetation index, NDVI) was employed to reflect the amount of plant cover at a specific location. High, middle, and low values of the vegetation index may refer to various growth stages of forest, grassland, and extremely low values refer to desert. Xiao et al. [15] reported that NDVI, temperature vegetation dryness index (TVDI), temperature, and precipitation significantly correlated with rodent density in Changsha City. Liu et al. [11] investigated the impacts of precipitation, temperature, land use types, compound topographic index, elevation, human footprint index, NDVI, eco-geographical characteristics, and distance to the nearest water sources on HFRS in Dongting Lake District, finding that NDVI and land use type were the top two dominant factors with attributable fraction of 62.7% and 7.9% HFRS cases, respectively.

In China, most previous studies quantifying the impacts of climatic factors or vegetation on HFRS incidence were conducted in a single-site or a few sites during a short-term period. Given that China is a climatically diverse country and the association between HFRS and environmental factors may be non-linear, small-scale analysis would make the studies from stepping into a heterogeneous relationship modeling and assessment difficult. The present study aims to use the advanced distributed lag non-linear model (DLNM) to assess the long-term impacts of temperature, rainfall, relative humidity, and NDVI on HFRS in 109 counties of China during a 12-year study period, which will help Chinese public health authorities to develop targeted prevention and control strategies for HFRS.

## 2. Materials and Methods

### 2.1. Data Collection

In this study we investigated 109 counties in China that represent the most important regions for HFRS outbreaks and where the highest numbers of cases have been reported. Data on monthly HFRS cases in the 109 counties from January 2002 to December 2013 were collected from the China Information System for Disease Control and Prevention (CISDCP). HFRS cases in each county were population-standardized using demographic data obtained from the National Bureau of Statistics of China (http://www.stats.gov.cn/). Climatic data, including mean temperature, rainfall, and relative humidity for the same study period, were collected from the China Meteorological Data Sharing Service System (http://data.cma.cn/). NDVI represents the vegetation lushness or vegetation coverage, which can reflect the agriculture biomass. Data on NDVI were extracted from the Free Vegetation Products with a temporal resolution of 10 days and served by VITO Belgium (http://www.vgt.vito.be/) and the NDVI data were digital values ranging from 0 to 255. During this period, the monthly HFRS cases, mean temperature, rainfall, relative humidity, and NDVI ranged from 0 to 312 cases, from −28.98 to 32.13 °C, from 0 to 704.84 mm, from 27.58 to 90.94%, and from 27.57 to 249.62, respectively (Figure S1).

### 2.2. Statistical Analysis

The impacts of mean temperature, rainfall, relative humidity, and NDVI on HFRS cases were assessed by employing a two-stage framework. Stage-I: time-series analysis was conducted to estimate the impact of each factor on HFRS cases at each county. Stage-II: the estimated effects in all counties were pooled at the national level of China for each factor using a random-effect meta-analysis.

#### 2.2.1. First Stage Analysis

At the first stage, to examine the exposure-response relationship with lagged effect for each county, a quasi-Poisson regression with a distributed lag non-linear model (DLNM) [18] was used to separately assess the impacts of the four factors on HFRS in each county. Because the range of climatic factors varied greatly in different counties, each factor was standardized to the percentile scale. Therefore, we assessed the relationships between climatic factors, vegetation, and HFRS based on a relative scale, following the methods previously described [19]. For each county, we modeled the non-linear and lagged effects of climatic factors using a cross-basis function of DLNM [18]. In this study, climatic factors and vegetation were fitted with natural cubic splines using four degrees of freedom and maximum lag time over four months. We set three internal knots at equally-spaced temperature percentiles (25th, 50th, and 75th) and two internal knots at equally spaced log transformed values of lag, respectively. The splines for climatic factors was centered at the percentile with the lowest risk of HFRS based on our preliminary analyses. The choice of four months for the lag period was motivated by the previous studies that showed how impacts of climatic factors mainly lasted for 3–5 months [5,15]. A natural cubic spline with three degrees of freedom per year for time was used to control for seasonality and long-term trend in the model.

#### 2.2.2. Second Stage Analysis

The coefficients of each factor were extracted from each county-specific exposure-lag-response association in the first stage. Then, we employed multivariate meta-analysis to pool the impact estimates of each factor [20,21]. The county-specific association was reduced to three summaries that expressed the overall cumulative exposure-response relationship and the lag-response relationships. For temperature, the lag-response relationship was defined as relative risk (RR) of HFRS at the 50th percentile of temperature comparing to minimum-HFRS temperature percentile (25th percentile). For rainfall, the lag-response relationship was defined as RR of HFRS at the 25th percentile of rainfall compared to a minimum-HFRS rainfall percentile (0th percentile). For relative humidity, the lag-response relationship was defined as RR of HFRS at the 25th percentile of relative humidity comparing to minimum-HFRS relative humidity percentile (0th percentile). For NDVI, the lag-response relationship was defined as RR of HFRS at the 50th percentile of NDVI comparing to minimum-HFRS NDVI percentile (25th percentile). At the first stage, the results from various counties may be heterogeneous. Then, a random-effect model with maximum likelihood was employed to fit multivariate meta-analysis at the second stage for synthesis analysis; and further the significance of meta-parameter and differences between various DLNM were tested by a multivariate extension of the likelihood ratio test [22].

#### 2.2.3. Sensitivity Analysis

Sensitivity analysis was conducted by changing maximum lag time of environmental factors from two to six months to see whether four months was adequate to capture the lagged effect of climatic factors and vegetation. In addition, we also modified the degrees of freedom (DF) of climatic factor and vegetation variables (4 to 5 DF) and time of the months (2 to 4 DF per year).

All data analysis was processed using R software (version 3.3.0, R Foundation for Statistical Computing, Vienna, Austria) with “dlnm” and “mnmeta” packages [23].

## 3. Results

**Figure 1:**
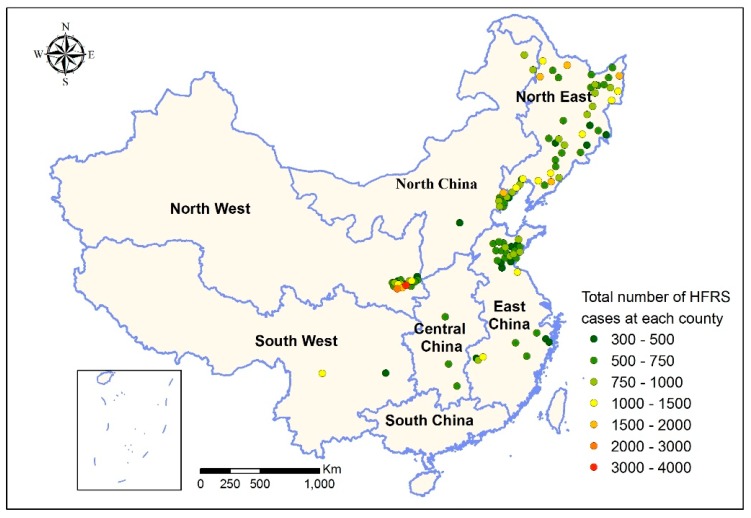
Total number of hemorrhagic fever with renal syndrome (HFRS) cases for the study period 2002–2013 in 109 counties of China.

A total of 88,830 HFRS cases in the 109 counties during the study period were extracted from CISDCP. Figure 1 shows the geographical distribution of the selected counties and the information on HFRS cases in these counties, suggesting that the counties in North East China and North West China had more HFRS cases than other regions during the study period. More descriptive statistics can be found in Figures S1 and S2. Moreover, a hot-spot analysis was employed in order to detect the spatial autocorrelation (including high HFRS cases clusters and low HFRS cases clusters) among the 109 counties of China. Due to the sparse distribution of the 109 counties, high HFRS cases clusters with 90% confidence are located in in the North West and low HFRS cases clusters with 90% and 95% confidence are located in East China (Figure S3).

**Figure 2:**
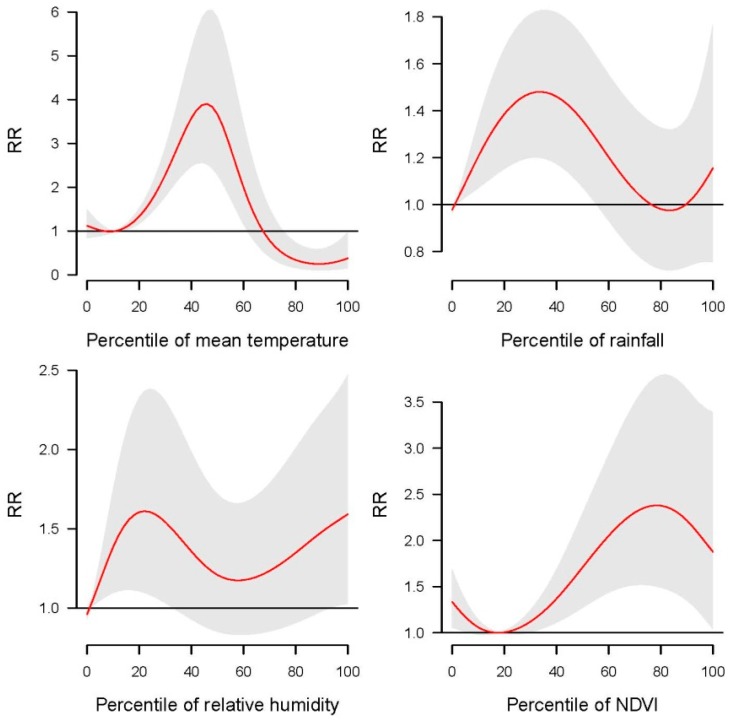
Pooled national level cumulative impacts of mean temperature, rainfall, relative humidity and normalized difference vegetation index (NDVI) on HFRS infection over lag 0-4 months during 2002–2013. RR represents relative risk.

Figure 2 shows the pooled dose-response relationships between climatic factors and HFRS cases of the 109 counties. The greatest relative risk (RR) of HFRS occurred when mean temperature, rainfall, relative humidity, and NDVI were at its 45th, 30th, 20th, and 80th percentiles, respectively. The real values of the four factors at its 45th, 30th, 20th, and 80th percentiles were different from each other. For illustration purpose, one typical high HFRS-outbreak county was selected from each of the six regions (i.e., North East, North China, North West, East China, Central China, South West); in the six selected counties, the real values of the four factors at its corresponding percentiles were shown in Figure S4. For example, in the Chang’an County from the North West, the real values of the four factors at corresponding percentiles were 13.08 °C, 18.01 mm, 58.37%, and 191.98, respectively, while the real values were 14.28 °C, 21.76 mm, 65.19%, and 193.72 in the Guanyuan County from East China. After calculating the real values of the four factors at its corresponding percentiles at each of the 109 counties, the mean real values with standard deviation of the four factors at corresponding percentiles were 10.58 ± 4.52 °C, 18.81 ± 17.82 mm, 58.61 ± 6.33%, and 198.20 ± 22.23. We also used the same procedure to explore the pooled dose-response relationships between climatic factors and HFRS cases in each sub-regions of China, i.e., North East, North China, North West, East China, Central China and South West. Particularly, the greatest RR of HFRS occurred at around 40th percentiles of mean temperature in North East China, East China, Central China, and North West China. In South West China, the greatest RR appeared at 80th percentiles of mean temperature (Figure S5). For rainfall, non-significant impacts (i.e., the values of log-transformed RR are smaller than zero in most percentiles of considered variable) were found in North East and North China, and the greatest impacts on HFRS were found at 20th, 65th, 80th, 20th percentiles, respectively, in East China, Central China, South West China and North West China (Figure S6). The impact of relative humidity on HFRS was the greatest at around 20th percentile in North East China, East China, and South West China, but negligible in North China and North West China (Figure S7). The greatest impact of NDVI on HFRS occurred at 45th, 80th, and 70th percentiles in North China, Central China, and North West China, respectively (Figure S8).

**Figure 3:**
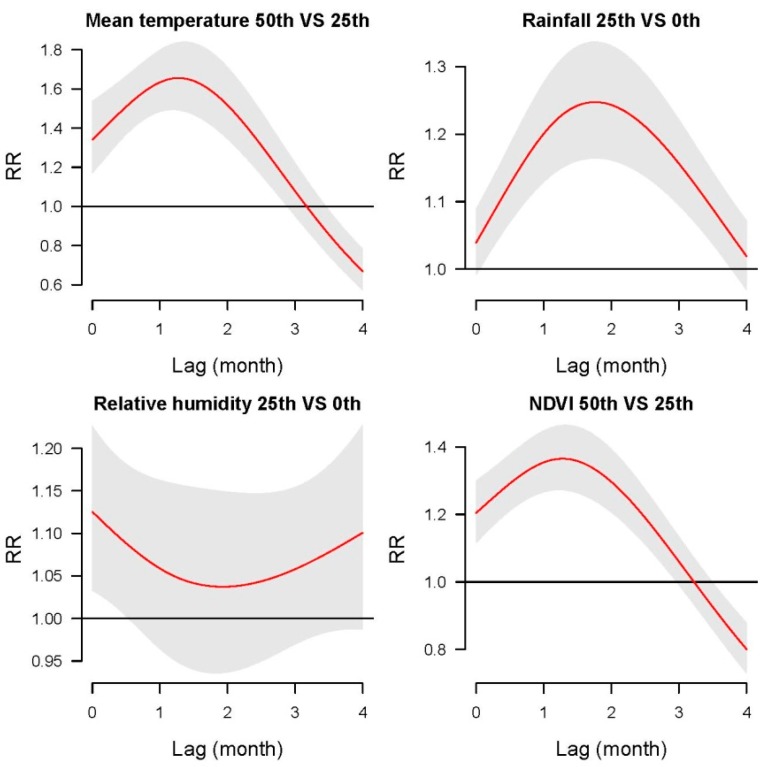
Pooled national level lagged impacts of mean temperature (50th percentile against 25th percentile), rainfall (25th percentile against 0th percentile), relative humidity (25th percentile against 0th percentile), and NDVI (50th percentile against 25th percentile) on HFRS infection from lag 1 to 4 months during 2002–2013.

Figure 3 shows the lagged effects of four factors on HFRS, including mean temperature (50th percentile vs. 25th percentile), rainfall (25th percentile vs. 0th percentile), relative humidity (25th percentile vs. 0th percentile), and NDVI (50th percentile vs. 25th percentile). The greatest impact of mean temperature and NDVI occurred in a lagged one-month and the greatest impact of rainfall happened in a lagged two-month. The impact of relative humidity on HFRS appeared in the current month.

**Figure 4:**
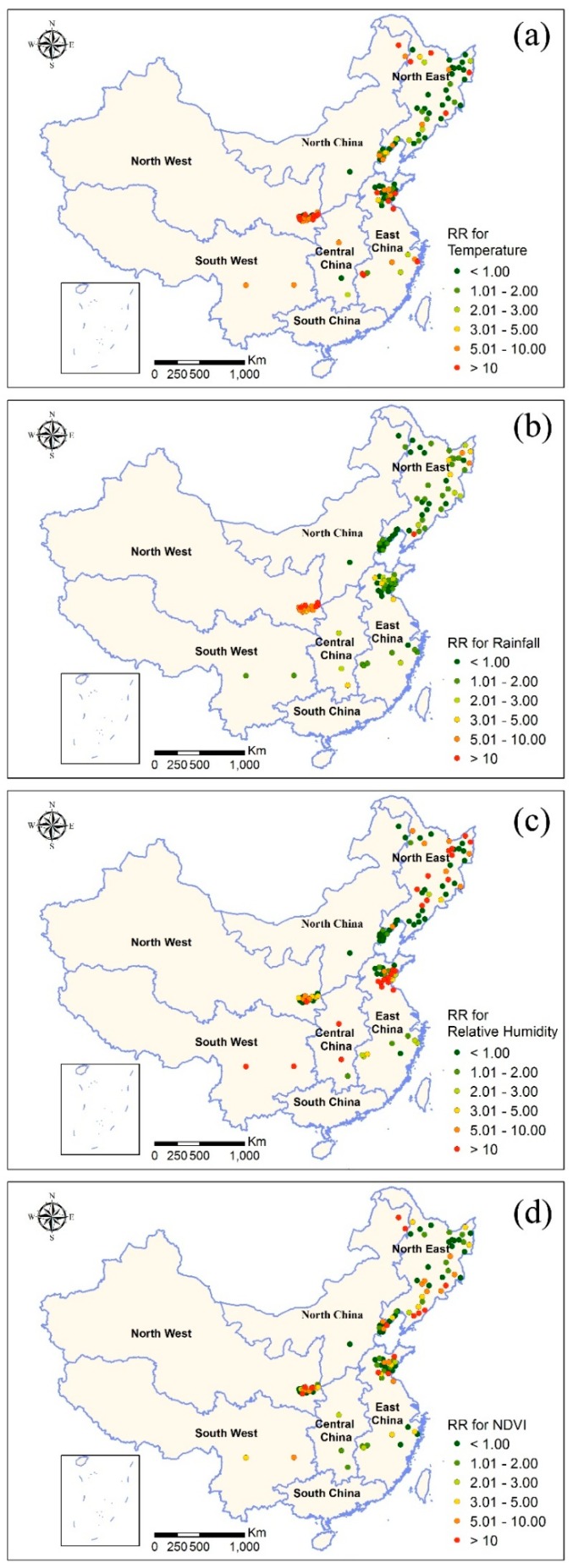
County-specific relative risk of (**a**) mean temperature (50th percentile against 25th percentile), (**b**) rainfall (25th percentile against 0th percentile), (**c**) relative humidity (25th percentile against 0th percentile), and (**d**) NDVI (50th percentile against 25th percentile) on HFRS infection.

Figure 4 shows the county-specific impacts of mean temperature, rainfall, relative humidity, and NDVI on HFRS cases, suggesting comparable geographical heterogeneities in the impacts of these climatic factors and NDVI on HFRS. HFRS infections in some counties from North West China and East China were more sensitive to mean temperature, rainfall, relative humidity, and NDVI (e.g., the RR for the climatic factors or vegetation were higher than 10). Particularly, the counties most sensitive to rainfall variation are located in North West China. In addition, sensitivity of the four factors on HFRS varied randomly in the other parts of China.

A sensitivity analysis showed that the results were reliable when changing the lag of month to three months, the DF of the four factors to 4 or 5 DF, and the DF of time to 1, 2, or 4 DF (Figures S9–S14).

## 4. Discussion

To the best of our knowledge, this is the first multi-county study to explore the impacts of climatic factors (mean temperature, rainfall, and relative humidity) and NDVI on HFRS in China. It has yielded four major findings: (i) non-linear associations between the considered four factors and HFRS were observed in this multi-county study. The greatest RR of the four factors on HFRS occurred at 45th, 30th, 20th, and 80th percentiles, respectively; (ii) among the four factors, HFRS was most sensitive to mean temperature; (iii) the lengths of lag time were heterogeneous, i.e., relative humidity (almost synchronous) < mean temperature (one-month lag) ≈ NDVI (one-month lag) < rainfall (two-month lag); (iv) the impact of rainfall on HFRS appeared to cluster in North West China. These findings can provide rigorous scientific support to current disease monitoring and early warning control procedures.

Multi-site studies on the relationship between climatic factors (or vegetation) and HFRS at a large spatial and temporal scale is still scarce in China. In this study, we tried to fill the gap by quantifying the impact of climatic factors and NDVI on HFRS during January 2002 and December 2013 in 109 Chinese counties. Using a novel approach with a two-stage analytical framework [22], it is possible to explore the heterogeneity of the relationship between climatic factors and disease relationship in multi-sites. This approach has been successfully applied in a multi-city analysis, quantifying temperature-emergency department visits, temperature-hospitalizations, temperature-mortality, ambient fine particles-influenza, temperature-childhood hand, and foot and mouth disease relationship [19,24,25,26,27]. This is first time this framework has been used and tested to explore the HFRS-climatic factors (or vegetation) associations in China.

Local environmental conditions can influence the human HFRS cases through affecting the transmission cycle of hantavirus, including virus activity, rodent populations, and transmission probability to humans [28,29,30,31]. Appropriate environmental conditions can promote the HFRS transmission. The present study unveiled the relationship between three climatic factors (or vegetation) and HFRS. Firstly, the impact of temperature on RR of HFRS displayed an increasing-decreasing trend, which can be explained by the physiology of rodent hosts. *Apodemus agrarius* and *Rattus norvegicus* have the highest breeding rate at a temperature range of 10–15 °C; the present study found the highest RR of HFRS occurred at 45 percentile of the mean temperature (i.e., 10.58 °C), which was rather close to the highest breeding temperature. Too cold or too hot weather negatively affect rodents’ activity and survival rate, preventing them from transmitting virus between infected donors and susceptible rodents or humans [5,6,29,32]. Secondly, an increasing-decreasing trend was found in the relationship between rainfall and RR of HFRS. A suitable amount of rainfall can accelerate the growth of foods, affecting the rodent populations [13,33,34,35]. However, floods caused by large amount of precipitation can not only damage the primary production of food but also the rodents’ burrows, restricting the reproduction of rodents. Thirdly, this study found that 20th percentile of the relative humidity was characterized with the greatest RR of HFRS. Compared to rainfall, relative humidity (sometimes subjected to runoff and rainfall) is another type of water source. Relative humidity can not only dominate the survival of viruses in the ambient atmosphere by influencing the physiology of virus but also influence the formation of aerosols that allows the viruses-transmission towards humans, especially in high-humidity environments [36]. Fourthly, our results in this study showed that high and low NDVI leads to low HFRS incidence, which is similar to the results obtained by Yan et al. [37]. NDVI represents the amount of vegetation coverage on ground. It can, to some extent, represent the amount of food provided to rodents. The NDVI values of the 109 counties range from 56.97 ± 22.74 to 225.12 ± 19.28 with different land cover (Figure S15). At the centroid of the 109 counties, 55, 24, 4, 20, and 6 of them are cropland, woodland, grassland, urban and barren, respectively. At a specific location, no matter what kind of land cover type (e.g., cropland, woodland, etc.) it is, the NDVI values will also vary a lot at different seasons due to the growth cycle of plants. Low NDVI values represent only a little amount of plant growth, where insufficient food is provided for rodents, leading to a low rodent population; on the contrary, higher NDVI values mean that the plants at the considered location grow better. It not only attracts more rodents to live in but also has large probability of human presence, because this kind of regions may be cropland with human working in them, which can provide shelters for rodents’ reproduction and increase the probability of human infection of HFRS [11,38]. However, in an extremely high NDVI region, the plants may be too dense for meeting the living conditions of the reservoir rodents.

Comparing Figure 3 with Figures S5–S8, we can find that the impacts of each factor on HFRS infection in North East, East China, and North West (see the curve shapes) dominate the cumulative impacts of the corresponding factor on HFRS infection on the national level. It means that the characteristics of these three sub-regions (including 92 counties out of the 109 counties) dominate the entire study area.

DLNM is flexible to explore the exposure-lag-response relationship in exposure-response dimension, but also lag-response dimension, i.e., the lagged effects of environmental factors on HFRS cases [27]. The lagged effects display the response time of virus development time, rodent growth, human infection, and virus incubation period [39]. Previous studies have found lag effects of temperature, rainfall, relative humidity, and NDVI on HFRS cases, incidence, and rodent density. The lagged effects of various environmental factors have different impacts on HFRS transmission (i.e., one-month lagged effects or above). These findings can only be utilized in a restrict area for further study. Moreover, some methods have mixed various lagged effects in the same model, which might cause a mode mixing problem. Considering this situation, this study employed DLNM, which can separated various lagged effects, so a dependent lagged effects from various time instance can be obtained. Further, meta-analysis can provide a synthesis tool to collect lagged effects on various location, which reflect a summary situations across a large spatial scale in China.

From an ecological view, relative humidity can directly interrupt the routine of virus spread from one rodent to another host (including human), as mentioned before. Therefore, the impact of relative humidity on HFRS is instantaneous without any lag. However, for the other three factors (i.e., rainfall, temperature, and vegetation), there exists an ecological chain, impacting HFRS transmission through influencing the rodent populations and furthering the probability of human exposure. Rainfall is the precipitation that falls onto the ground, providing water sources for plant growth, especially primary production; it can be served as food for rodents’ growth and reproduction. Meanwhile, temperature presents living conditions for rodents’ growth and reproduction. In other words, these three factors impact HFRS transmission indirectly; maximal lagged effects of rainfall display at two months, but NDVI and temperature at one-month. Understanding the lagged effects is helpful for disease control managers to make decisions, take measures and implement interventions, such as improving surveillance system, warning and educating high risk groups of human to reduce their risk through environmental modification and hygiene practices, adjusting allocation of vaccine, encouraging vaccination, widely culling rodents, and cleaning the wild environment [5,40]. Moreover, Figure 4 displayed the local RR of four environmental factors on HFRS. Through comparison among the four maps, a dominant factor at each county can be figured out, so the local disease control and prevention management should pay more attention to this factor. We believe that the generated RR (single parameter) at each county in Figure 4 can, to some extent, be used for HFRS-early warning and HFRS-controlling decision making. However, the feasibility of applying the single parameter should be evaluated in future; further, RR together with other HFRS risk assessments should be explored for constructing a comprehensive HFRS-early warning system. Globally, these maps can be used to develop appropriate strategies and optimize the health resources distribution for HFRS control and prevention. For instance, more concerns and public health resources should be set into high RR areas.

Limitations of the present study should be acknowledged. Firstly, monthly HFRS data was, to some extent, coarse for analysis; weekly data has been successfully used in exploring the climatic factor-HFRS relationship [41,42], which is more precise for HFRS early warning than monthly data. Secondly, rodent populations are one of the factors, which determine the probability of human infection. It constructs the connection between HFRS virus and human infections, i.e., transmission of HFRS; hence, study the impacts of environmental factors on rodent populations can provide more information in HFRS early warning and prevention. Thirdly, the HFRS data was extracted from a passive surveillance system; we cannot distinguish the various types of HFRS cases, e.g., *Hantaan* orthohantavirus and *Seoul* orthohantavirus agents induced HFRS cases.

## 5. Conclusions

This study quantified the impacts of environmental factors on HFRS in China. This study’s findings can contribute to a better understanding of the ecology of HFRS in China and may help develop the HFRS early warning systems, providing public health authorities in China for developing effective prevention and control strategies for HFRS. The employed methodology framework in the current study can be also applied when exploring the HFRS-environmental factors associations in other countries or other diseases.

## Supplementary Materials

The following are available online at https://www.mdpi.com/1660-4601/16/18/3434/s1, Figure S1: Histograms and descriptive statistics of the HFRS cases, mean temperature, rainfall, relative humidity and NDVI in the 109 counties of China during the period from January 2002 to December 2013 (*N* = 15696), Figure S2: Annual mean (a) temperature, (b) rainfall, (c) relative humidity and (d) NDVI in 109 counties of China during the period from January 2002 to December, Figure S3: Hot-spot analysis of the total HFRS cases in the 109 counties of China during the period from January 2002 to December, Figure S4: The real values of the mean temperature, rainfall, relative humidity and NDVI at its 45th, 30th, 20th and 80th percentiles in the six selected counties, Figure S5: Pooled multi-county level cumulative impacts of mean temperature on HFRS infection over 0–4 months lag during the period from January 2002 to December, Figure S6: Pooled multi-county level cumulative impacts of rainfall on HFRS infection over 0–4 months lag during the period from January 2002 to December, Figure S7: Pooled multi-county level cumulative impacts of relative humidity on HFRS infection over 0–4 months lag during the period from January 2002 to December, Figure S8: Pooled multi-county level cumulative impacts of NDVI on HFRS infection over 0–4 months lag during the period from January 2002 to December, Figure S9: Pooled national level cumulative impacts of mean temperature, rainfall, relative humidity and NDVI on HFRS infection (left) and pooled national level lagged impacts of the four factors on HFRS infection along 0–4 months during 2002–2013 (right) by setting the time lag as 3 months, Figure S10: Pooled national level cumulative impacts of mean temperature, rainfall, relative humidity and NDVI on HFRS infection (left) and pooled national level lagged impacts of the four factors on HFRS infection along 0–4 months during 2002–2013 (right) by setting the degrees of freedom (DF) of the four factors to 4 DF, Figure S11: Pooled national level cumulative impacts of mean temperature, rainfall, relative humidity and NDVI on HFRS infection (left) and pooled national level lagged impacts of the four factors on HFRS infection along 0–4 months during 2002–2013 (right) by setting the degrees of freedom (DF) of the four factors to 5 DF, Figure S12: Pooled national level cumulative impacts of mean temperature, rainfall, relative humidity and NDVI on HFRS infection (left) and pooled national level lagged impacts of the four factors on HFRS infection along 0–4 months during 2002–2013 (right) by setting the degrees of freedom (DF) of the time as 1 DF, Figure S13: Pooled national level cumulative impacts of mean temperature, rainfall, relative humidity and NDVI on HFRS infection (left) and pooled national level lagged impacts of the four factors on HFRS infection along 0–4 months during 2002–2013 (right) by setting the degrees of freedom (DF) of the time as 2 DF, Figure S14: Pooled national level cumulative impacts of mean temperature, rainfall, relative humidity and NDVI on HFRS infection (left) and pooled national level lagged impacts of the four factors on HFRS infection along 0–4 months during 2002–2013 (right) by setting the degrees of freedom (DF) of the time as 4 DF, Figure S15: Land cover type at the centroid of administrative boundary in each of the 109 counties, Table S1: Descriptive statistics of mean temperature, rainfall, relative humidity and NDVI in 109 counties (Table S1 is in a XLS file named “Table S1”).
