# Supplementary material for: The Impacts of Climatic Factors and Vegetation on Hemorrhagic Fever with Renal Syndrome Transmission in China: A Study of 109 Counties
Source: Int J Environ Res Public Health. 2019 Sep 16;16(18):3434. doi: 10.3390/ijerph16183434 (PMC6765884; doi:10.3390/ijerph16183434)
Supplement: Supplementary file 1 [file ijerph-16-03434-s001.zip › 0916 HFRS-SI.pdf]

Supplementary Materials

Figure S1 Histograms and descriptive statistics of the HFRS cases, mean temperature, rainfall, relative humidity and NDVI in the 109 counties of China during the period from Jan. 2002 to Dec. 2013 (N = 15696)
Figure S2 Annual mean (a) temperature, (b) rainfall, (c) relative humidity and (d) NDVI in 109 counties of China during the period from Jan. 2002 to Dec. 2013
Figure S3 Hot-spot analysis of the total HFRS cases in the 109 counties of China during the period from Jan. 2002 to Dec. 2013
Figure S4 The real values of the mean temperature, rainfall, relative humidity and NDVI at its 45th, 30th, 20th and 80th percentiles in the six selected counties
Figure S5 Pooled multi-county level cumulative impacts of mean temperature on HFRS infection over 0-4 months lag during the period from Jan. 2002 to Dec. 2013
Figure S6 Pooled multi-county level cumulative impacts of rainfall on HFRS infection over 0-4 months lag during the period from Jan. 2002 to Dec. 2013
Figure S7 Pooled multi-county level cumulative impacts of relative humidity on HFRS infection over 0-4 months lag during the period from Jan. 2002 to Dec. 2013
Figure S8 Pooled multi-county level cumulative impacts of NDVI on HFRS infection over 0-4 months lag during the period from Jan. 2002 to Dec. 2013
Figure S9 Pooled national level cumulative impacts of mean temperature, rainfall, relative humidity and NDVI on HFRS infection (left) and pooled national level lagged impacts of the four factors on HFRS infection along 0-4 months during 2002-2013 (right) by setting the time lag as 3 months
Figure S10 Pooled national level cumulative impacts of mean temperature, rainfall, relative humidity and NDVI on HFRS infection (left) and pooled national level lagged impacts of the four factors on HFRS infection along 0-4 months during 2002-2013 (right) by setting the degrees of freedom (DF) of the four factors to 4 DF
Figure S11 Pooled national level cumulative impacts of mean temperature, rainfall, relative humidity and NDVI on HFRS infection (left) and pooled national level lagged impacts of the four factors on HFRS infection along 0-4 months during 2002-2013 (right) by setting the degrees of freedom (DF) of the four factors to 5 DF
Figure S12 Pooled national level cumulative impacts of mean temperature, rainfall, relative humidity and NDVI on HFRS infection (left) and pooled national level lagged impacts of the four factors on HFRS infection along 0-4 months during 2002-2013 (right) by setting the degrees of freedom (DF) of the time as 1 DF
Figure S13 Pooled national level cumulative impacts of mean temperature, rainfall, relative humidity and NDVI on HFRS infection (left) and pooled national level lagged impacts of the four factors on HFRS infection along 0-4 months during 2002-2013 (right) by setting the degrees of freedom (DF) of the time as 2 DF
Figure S14 Pooled national level cumulative impacts of mean temperature, rainfall, relative humidity and NDVI on HFRS infection (left) and pooled national level lagged impacts of the four factors on HFRS infection along 0-4 months during 2002-2013 (right) by setting the degrees of freedom (DF) of the time as 4 DF
Figure S15 Land cover type at the centroid of administrative boundary in each of the 109 counties

Table S1 Descriptive statistics of mean temperature, rainfall, relative humidity and NDVI in 109 counties (Table S1is in a XLS file named "Table S1")

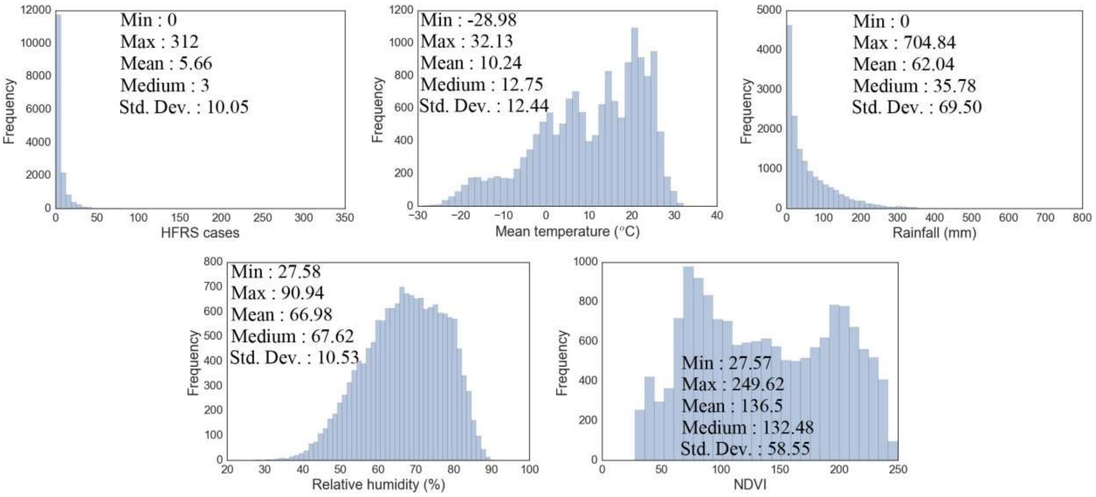

Figure S1. Histograms and descriptive statistics of the HFRS cases, mean temperature, rainfall, relative humidity and NDVI in the 109 counties of China during the period from Jan. 2002 to Dec. 2013 (N = 15696).

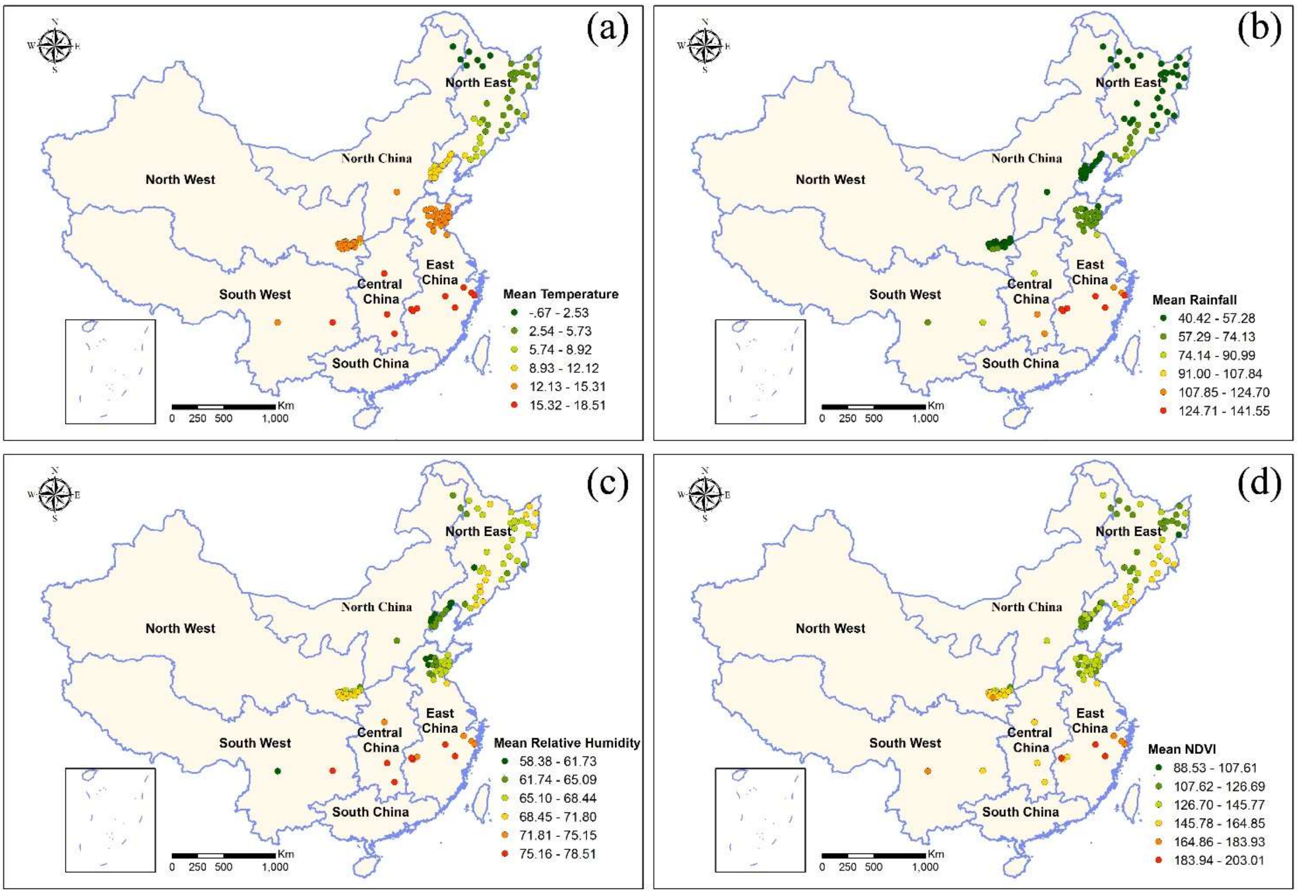

Figure S2. Annual mean (a) temperature, (b) rainfall, (c) relative humidity and (d) NDVI in 109 counties of China during the period from Jan. 2002 to Dec. 2013.

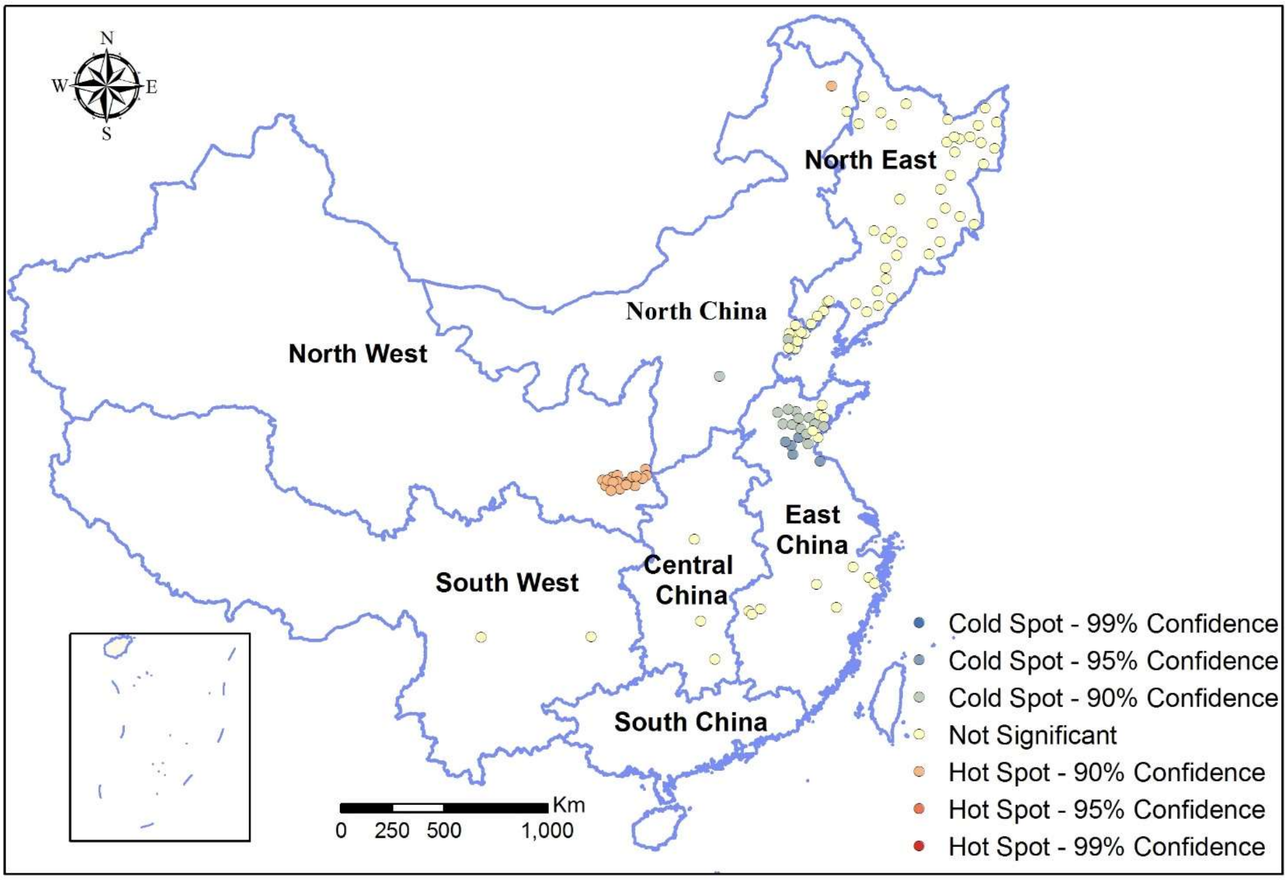

Figure S3. Hot-spot analysis of the total HFRS cases in the 109 counties of China during the period from Jan. 2002 to Dec. 2013.

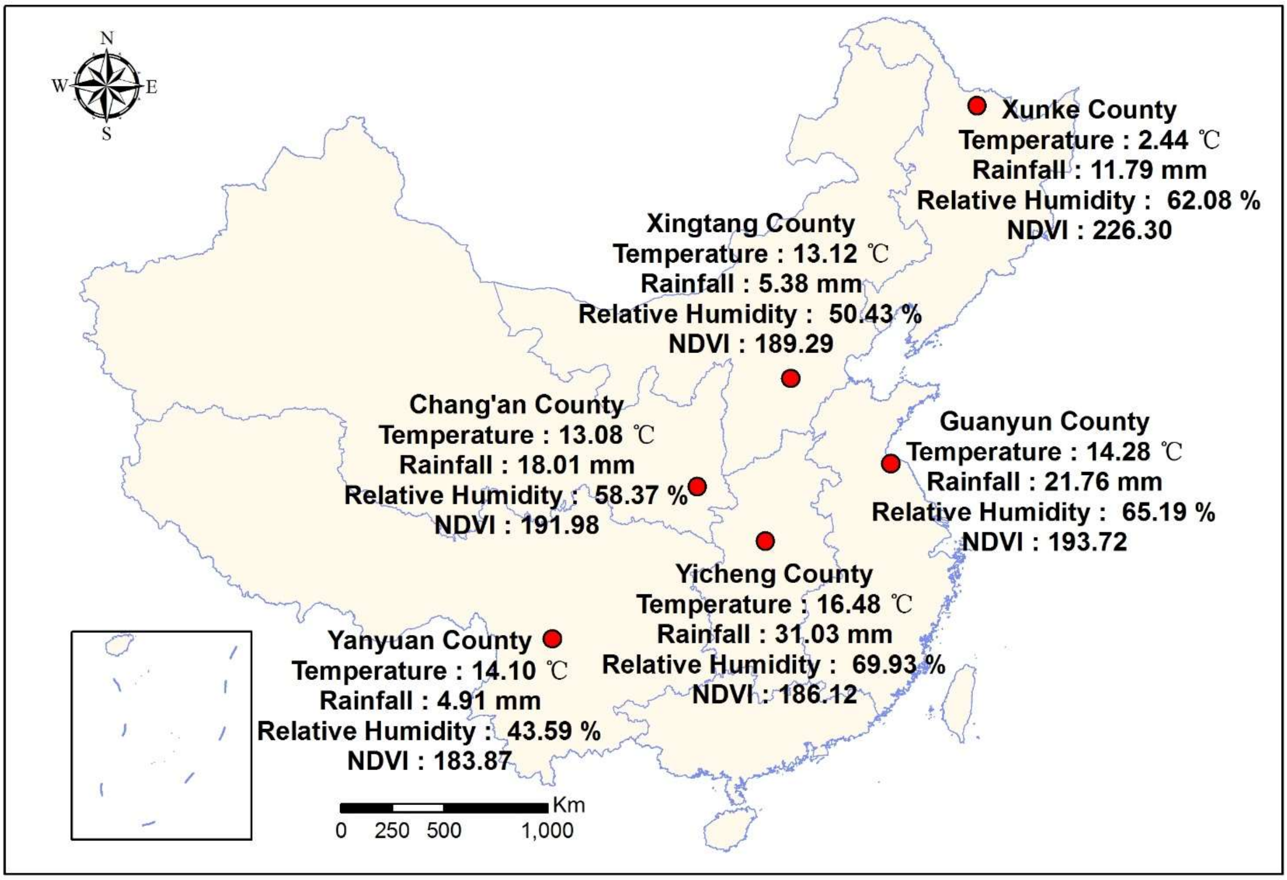

Figure S4. The real values of the mean temperature, rainfall, relative humidity and NDVI at its 45th, 30th, 20th and 80th percentiles in the six selected counties.

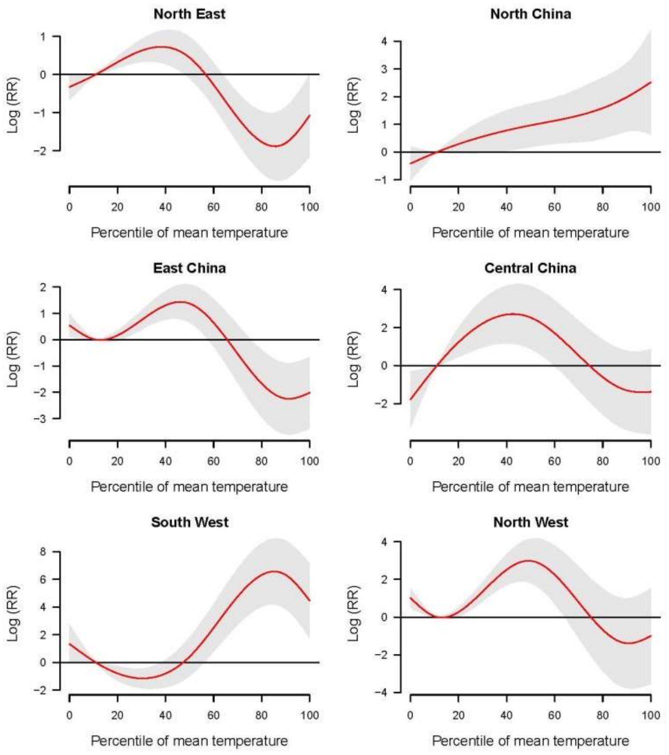

Figure S5. Pooled multi-county level cumulative impacts of mean temperature on HFRS infection over 0-4 months lag during the period from Jan. 2002 to Dec. 2013.

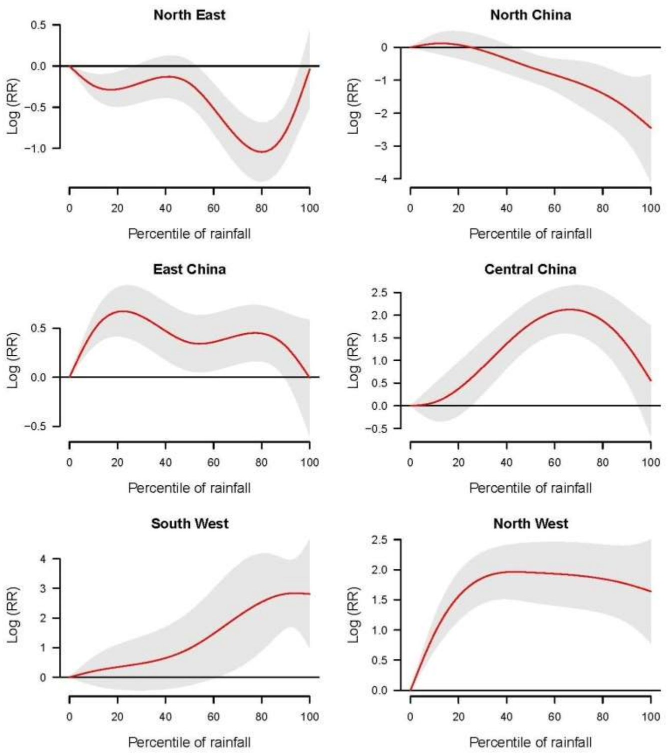

Figure S6. Pooled multi-county level cumulative impacts of rainfall on HFRS infection over 0-4 months lag during the period from Jan. 2002 to Dec. 2013.

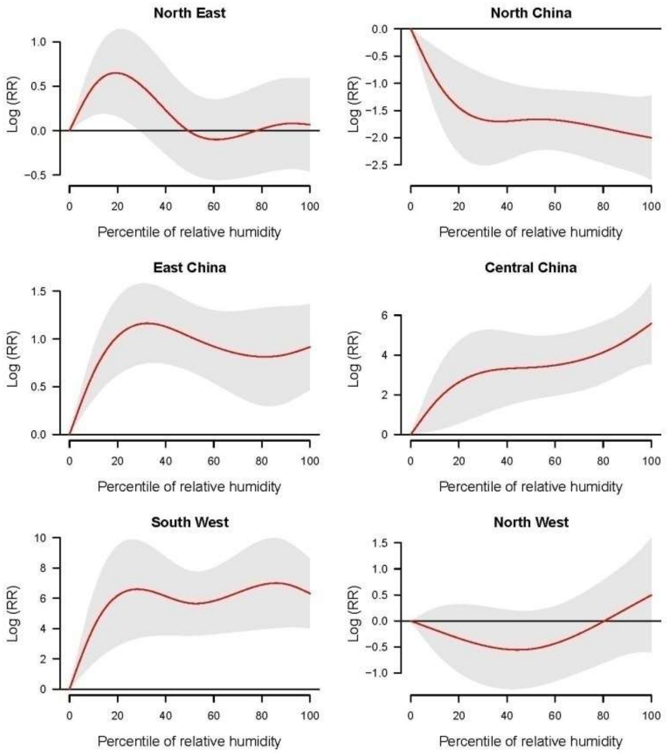

Figure S7. Pooled multi-county level cumulative impacts of relative humidity on HFRS infection over 0-4 months lag during the period from Jan. 2002 to Dec. 2013.

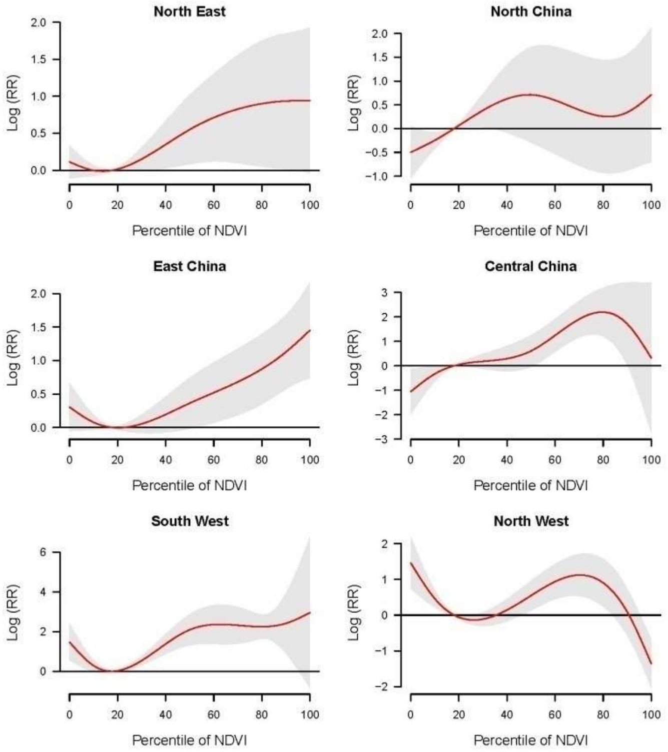

Figure S8. Pooled multi-county level cumulative impacts of NDVI on HFRS infection over 0-4 months lag during the period from Jan. 2002 to Dec. 2013.

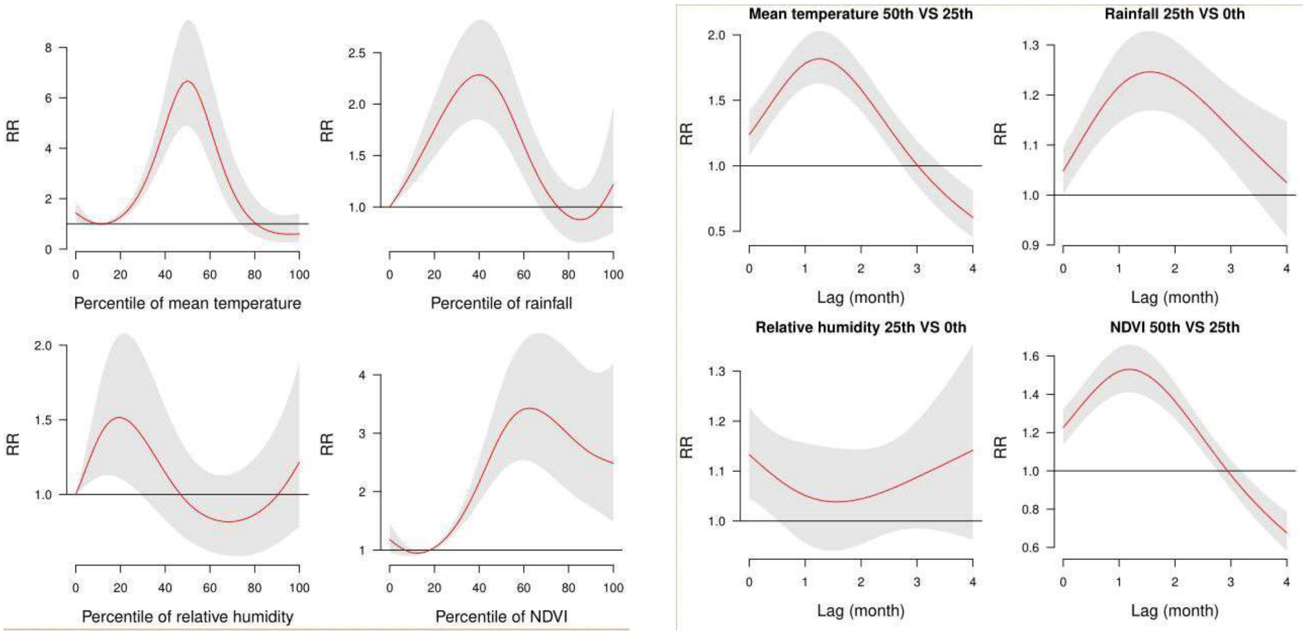

Figure S9. Pooled national level cumulative impacts of mean temperature, rainfall, relative humidity and NDVI on HFRS infection (left) and pooled national level lagged impacts of the four factors on HFRS infection along 0-4 months during 2002-2013 (right) by setting the time lag as 3 months.

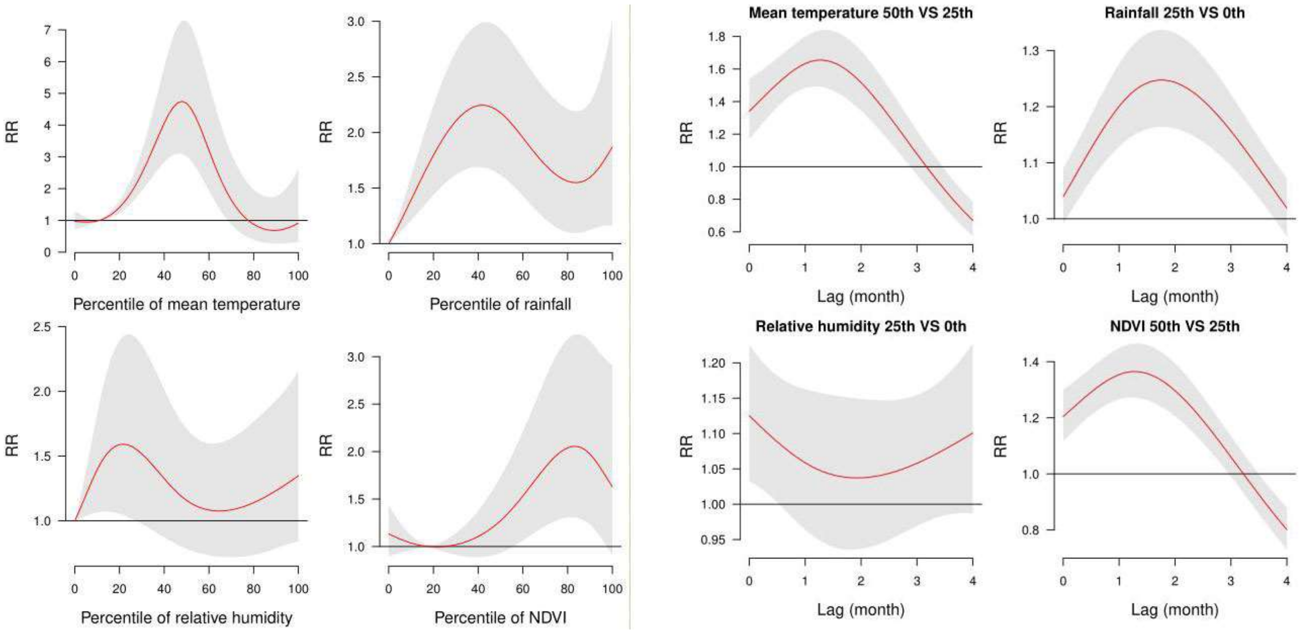

Figure S10. Pooled national level cumulative impacts of mean temperature, rainfall, relative humidity and NDVI on HFRS infection (left) and pooled national level lagged impacts of the four factors on HFRS infection along 0-4 months during 2002-2013 (right) by setting the degrees of freedom (DF) of the four factors to 4 DF.

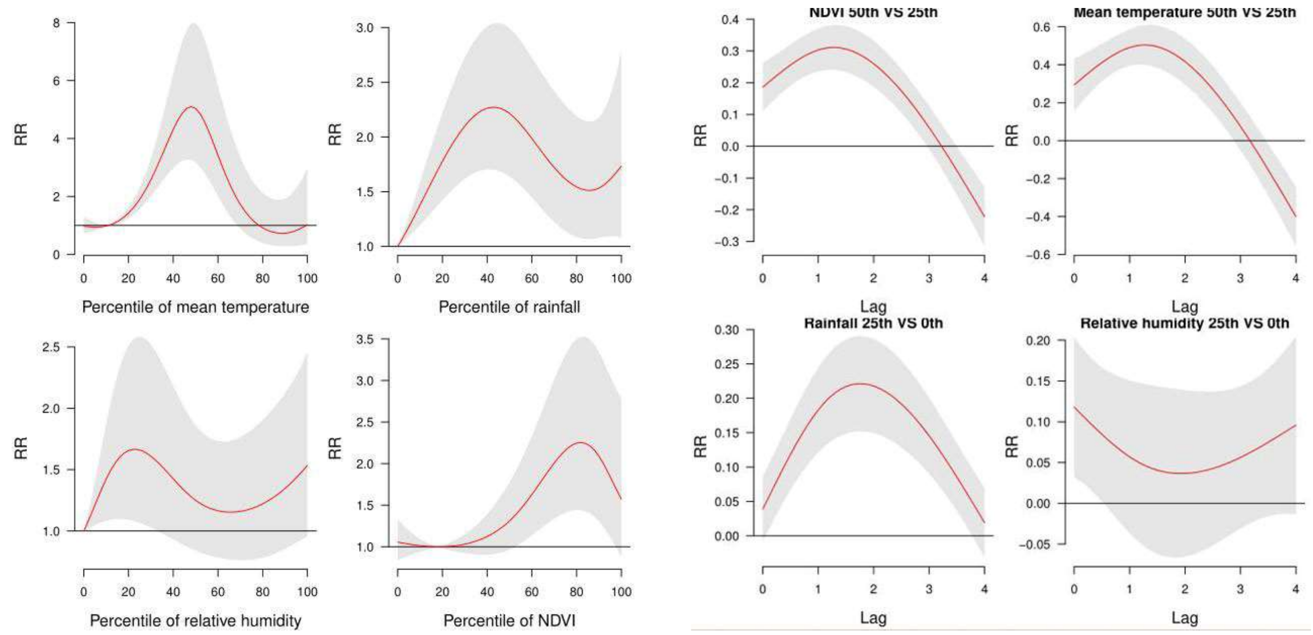

Figure S11. Pooled national level cumulative impacts of mean temperature, rainfall, relative humidity and NDVI on HFRS infection (left) and pooled national level lagged impacts of the four factors on HFRS infection along 0-4 months during 2002-2013 (right) by setting the degrees of freedom (DF) of the four factors to 5 DF.

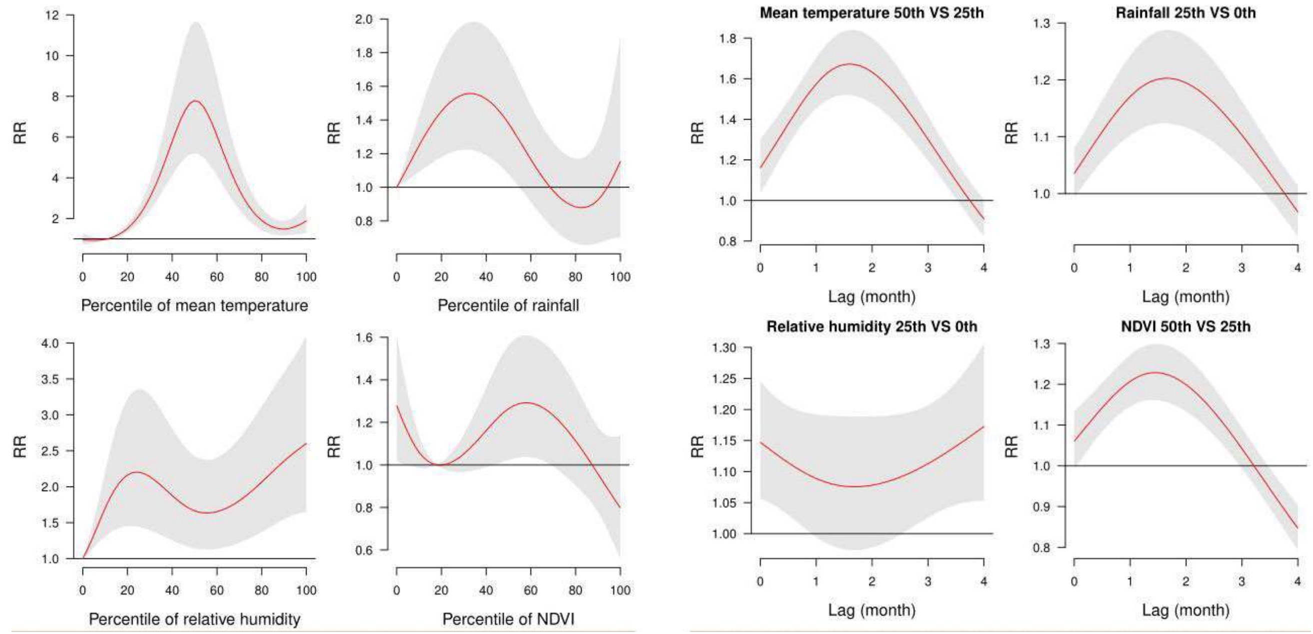

Figure S12. Pooled national level cumulative impacts of mean temperature, rainfall, relative humidity and NDVI on HFRS infection (left) and pooled national level lagged impacts of the four factors on HFRS infection along 0-4 months during 2002-2013 (right) by setting the degrees of freedom (DF) of the time as 1 DF.

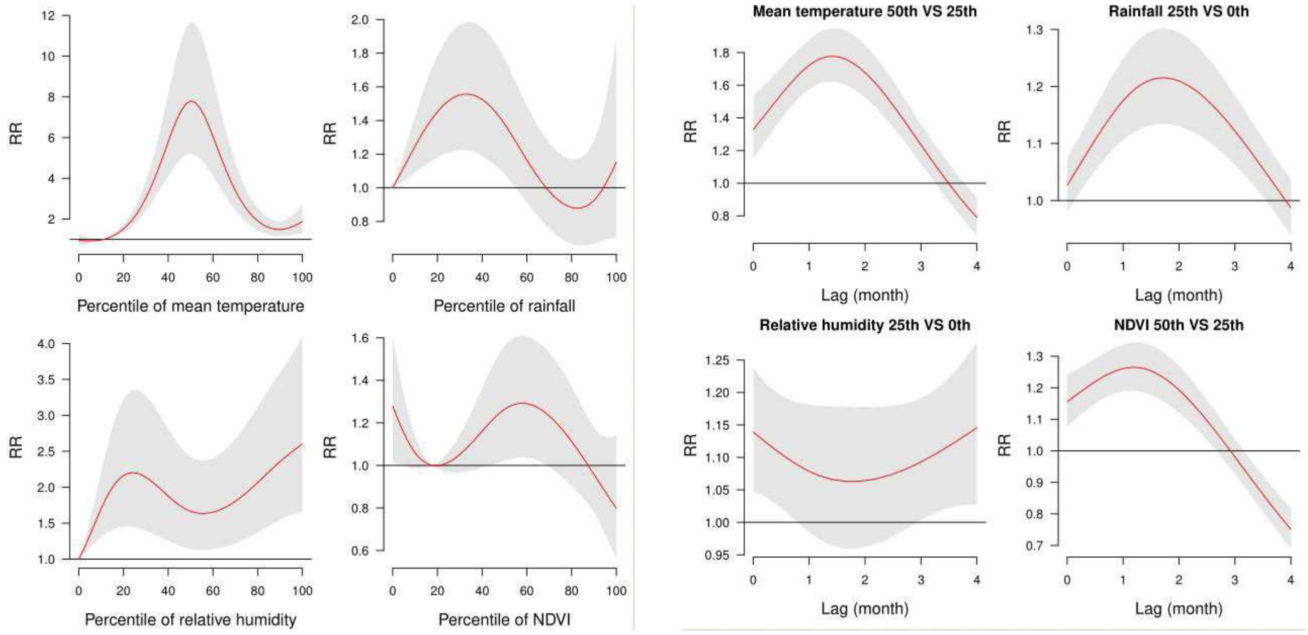

Figure S13. Pooled national level cumulative impacts of mean temperature, rainfall, relative humidity and NDVI on HFRS infection (left) and pooled national level lagged impacts of the four factors on HFRS infection along 0-4 months during 2002-2013 (right) by setting the degrees of freedom (DF) of the time as 2 DF.

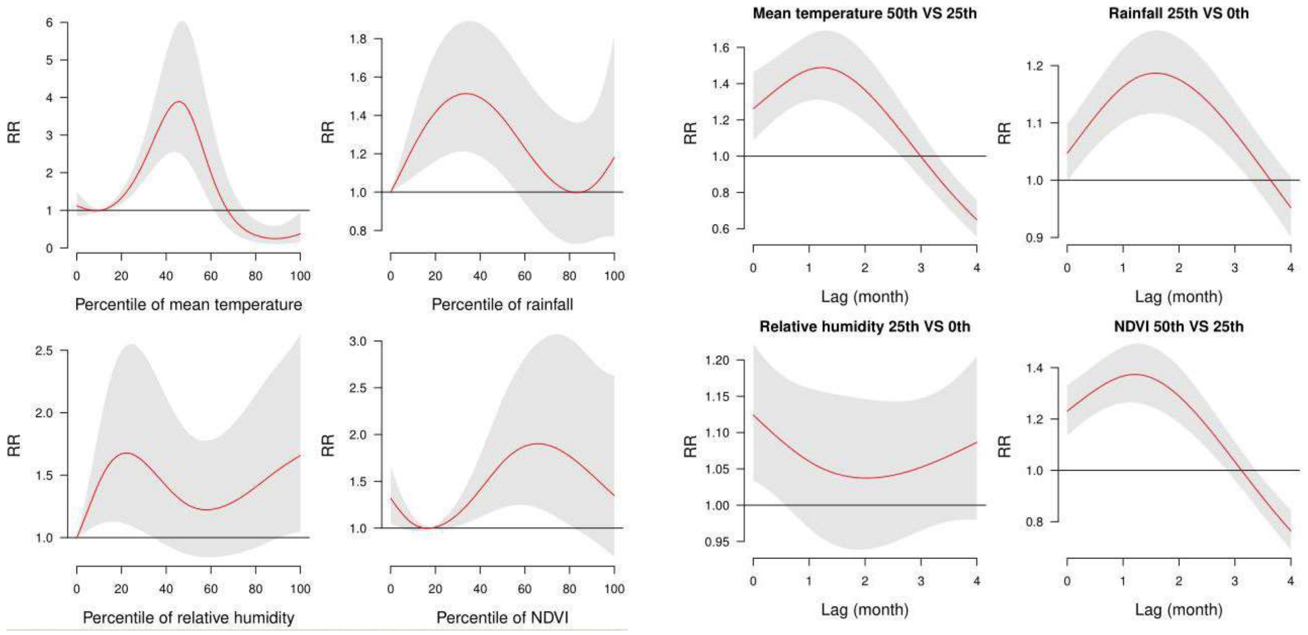

Figure S14. Pooled national level cumulative impacts of mean temperature, rainfall, relative humidity and NDVI on HFRS infection (left) and pooled national level lagged impacts of the four factors on HFRS infection along 0-4 months during 2002-2013 (right) by setting the degrees of freedom (DF) of the time as 4 DF.

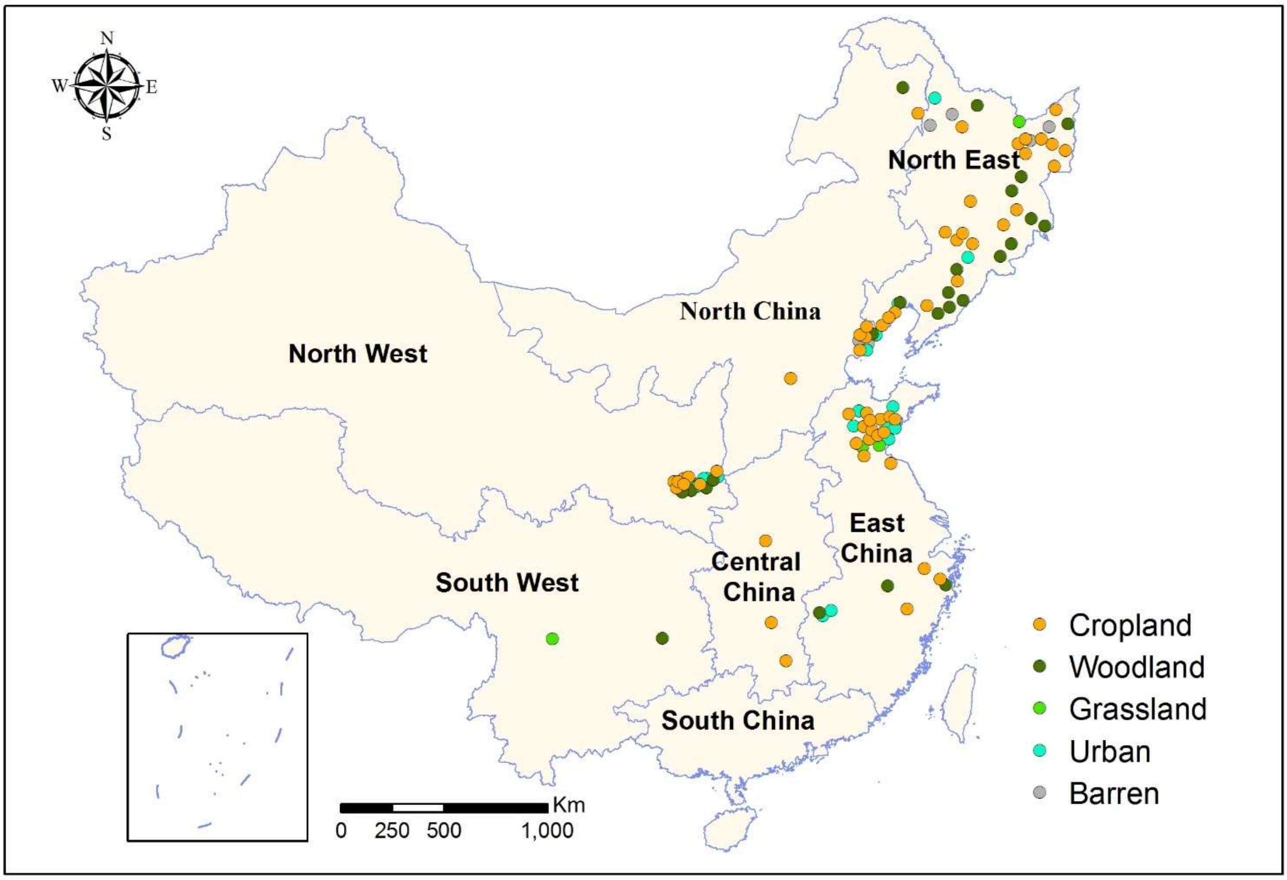

Figure S15. Land cover type at the centroid of administrative boundary in each of the 109 counties.

Table S1 Descriptive statistics of mean temperature, rainfall, relative humidity and NDVI in 109 counties (Table S1is in a .xlsx file named "Table S1").
